# Investigating the effect of vitamin D vaginal suppository on sexual function among postmenopausal women: study protocol for a randomized controlled trial

**DOI:** 10.1186/s12905-020-00899-6

**Published:** 2020-02-18

**Authors:** Zinat Sarebani, Zainab Alimoradi, Ehsan Aali, Monirsadat Mirzadeh, Venus Chegini, Mohammadreza Abbaspour, Mark D. Griffiths

**Affiliations:** 1 Students research committee, School of Nursing & Midwifery, Qazvin University of Medical Sciences, Qazvin, Iran; 2 Social Determinants of Health Research Center, Research Institute for Prevention of Non-Communicable Diseases, Qazvin University of Medical Sciences, Bahonar blv., Qazvin, 34197-59811 Iran; 3 Pharmacology Department, School of Medicine, Qazvin University of Medical Sciences, Qazvin, Iran; 4 Community Medicine Department, Metabolic Diseases Research Center, Research Institute For Prevention Of Non-Communicable Diseases, Qazvin University of Medical Sciences, Qazvin, Iran; 5 Obstetrics and Gynecology Department, School of Medicine, Qazvin University of Medical Sciences, Qazvin, Iran; 6 Targeted Drug Delivery Research Center, Pharmaceutical Technology Institute, Mashhad University of Medical Sciences, Mashhad, Iran; 7 Psychology Department, Nottingham Trent University, Nottingham, UK

**Keywords:** Menopause, Sexual function, Vitamin D vaginal suppository

## Abstract

**Background:**

Menopause is associated with changes in sexual function which are partly due to vaginal atrophy in response to estrogen reduction. Estrogen administration temporarily reduces the symptoms of vaginal dryness, but long-term exposure to this drug is likely to be associated with serious complications. Considering the promising results of previous studies concerning the effect of vitamin D on vaginal dryness, the proposed study will investigate the effect of vitamin D vaginal suppository on the sexual function of postmenopausal women.

**Methods:**

In a randomized, controlled clinical trial, 105 postmenopausal women will be randomly assigned to three groups receiving vitamin D vaginal suppository, placebo vaginal suppository, or control (no intervention). Vitamin D vaginal suppositories contain 1000 units of vitamin D3. The timing of the use of vitamin D vaginal suppositories and placebo suppositories will be every night in the first 2 weeks, and every other night in the following 6 weeks (8 weeks in total). The primary outcome will be the sexual function of participants which will be assessed using the Female Sexual Function Index (FSFI) before and immediately after the intervention, and at 1 and 2 months after the end of the intervention. The side effects of these suppositories will be examined as a secondary consequence of the study. Data will be analyzed using SPSS software version 25. In the case of normal distribution of data, the mean score of sexual function will be compared between the groups using a repeated measurements ANOVA. If statistical analysis leads to significant results, the post-hoc test will be used to determine the differences between the groups. Comparison of demographic and fertility characteristics of the women will be carried out using statistical tests such as chi-squares and *t*-tests. A significance level of *p* < .05 will be used for statistical analyses.

**Discussion:**

If vitamin D vaginal suppositories improve sexual function among premenopausal women with long-term effects and minimum side effects, the suppositories will be considered a safe complementary and alternative choice for alleviating sexual dysfunction among this group.

**Trial registration:**

IRCT20180704040346N1 at 2018-10-13 prospectively registered.

## Background

Menopause – as one of the critical and inevitable stages of women’s life – is the most important event during middle age. It can have a particular influence in women’s personal, cultural, social, reproductive health, and sexuality [1]. Menopause leads to many physiological changes that occur continuously and gradually, and can affect the woman’s life [2]. Menopause occurs gradually from the late third decade of life between the ages of 40 and 60 years and a mean of 51 years [1]. According to studies conducted in Iran, the range of menopausal age ranges between 46 and 52 years [3].

The climacteric phase and menopause are the periods of reductions in the production of hormones, that can affect women’s quality of life because of various complications [4]. These complications can include symptoms such as hot flushes, night sweats, palpitations, bone loss, urinary problems, and vaginal dryness [5]. Vaginal dryness (as one of the problems caused by the reduction of estrogen production) is due to thinning of the endometrial tissue and increase of pH, that cause local itching, an increase in the incidence of vaginal infection, and vaginal structural weakness. In addition, dyspareunia can cause sexual dysfunction and loss of sexual desire during menopause [6]. Vaginal atrophy, after hot flushes, is the second most common complication and is the worst complication of menopause [4]. Reducing estrogen levels through causing fractures in collagen and elastin fibers in the vagina causes vaginal atrophy. From a cytomorphological aspect, parabasal cells are increased and intermediate and superficial cells are decreased [7].

Problems with vaginal atrophy are clinically manifested 4–5 years after the beginning of menopause [8]. Vulvovaginal atrophy lead to vaginal dryness, irritation, soreness, and consequently dyspareunia [9, 10]. Approximately one-third of postmenopausal women report painful intercourse (dyspareunia), lack of moisture, and decreased lubrication [11]. Common symptoms of atrophy include vaginal dryness, burning, post-coital bleeding, and pain [12]. In clinical examination, symptoms such as being pale, the reduction of transverse vaginal folds (roga), petechiae, reduced elasticity, and dryness confirms the presence of atrophy [5]. Vaginal atrophy is one of the causes of sexual problems, and is one of the most important complaints among women during the menopause, but despite many advances in the prevention and treatment of menopausal complications, sexual problems are underestimated [13].

One of the therapeutic methods for improving the symptoms of vaginal atrophy and dyspareunia in postmenopausal women is estrogen therapy [14]. Estrogen improves the mucosa, and increases elasticity and blood flow to vulva and vaginal area. It also increases the sensory threshold of the vulva region and vagina, increases sexual pleasure, and improves arousal [15]. Since estrogen deficiency is the main cause of vaginal atrophy, estrogen therapy after menopause is the most rational choice of treatment. Systemic estrogen therapy is the gold standard in slowing down and/or preventing early genital atrophy, but maintaining its effect requires continuous treatment [2]. The other initial and preferable and choice in treating genitourinary syndrome of menopausal women is ultralow-dose topical estriol [16]. Overall, due to the potential risks of postmenopausal systemic hormone replacement therapy, estrogen replacement therapy may not be acceptable by many women [8]. Concerns about the complications of estrogen therapy, including cardiovascular events, thromboembolism, breast cancer, and endometrial hyperplasia are the most important reasons for the low acceptance of synthetic estrogen therapy [17]. In addition to the aforementioned concerns, a considerable proportion of menopausal women experience some type of chronic disease such as diabetes, cardiovascular disease, dyslipidemia, and asthma [18], so they are not good candidates when administering many kinds of hormone therapy. Regarding these issues, there is an increasing tendency to utilize alternative treatments for relieving menopausal symptoms [17].

Vitamin D is one of the essential substances in metabolic and physiological processes in the body [19]. Recent literature has reported the role of low level of vitamin D in many pathological conditions including cardiovascular disease, type 2 diabetes mellitus, metabolic syndrome, cancer, and increased mortality, as well as its role in calcium and bone metabolism [20]. Recent research has also shown that vitamin D3 may be useful in preventing vaginal atrophy. Vitamin D3 may play a role in regulating the growth and differentiation of the vaginal epithelium. Costantino’s research found that taking vitamin D3 can help prevent osteoporosis and eliminate vaginal discomfort after the menopause [6]. Vitale et al. (2018) reported after a randomized, placebo-controlled trial, that oral supplements of vitamin D in combination with isoflavones, calcium, and inulin significantly improved the sexual function of menopausal women [21]. In a cross-sectional study, Yildirmal et al. (2004) showed that the use of vitamin D supplements was effective in the maturation of vaginal cells [22]. Also, a clinical trial by Zainlugl et al. (2007) showed that in postmenopausal women with osteoporosis, Raloxifene and vitamin D supplementation significantly reduced vaginal dryness and pH [23].

Vitamin D3 can be absorbed in the vagina by applying a vaginal suppository [11]. The results of a clinical trial study by Rad et al. (2014) on postmenopausal women in Ahwaz (Iran) showed that vitamin D suppositories could experimentally (according to the results of the participants’ Pap smear) improve dryness and cell proliferation of the vaginal mucosa among postmenopausal women [24]. The possible mechanism for the effect of vitamin D on the vagina is due to the presence of intracellular receptors of this vitamin in the basal and parabasal cellular layer in the vaginal tissue. Because of these receptors in the vagina, vitamin D can play an important role in regulating and increasing the proliferation of epithelium cells in vagina [25, 26]. However, vitamin D receptors change during the menstrual cycle, which means that by stopping ovarian activity, the number of receptors are decreased [27].

The results of experiments in rats have shown that ovarian harvesting results in the loss of vitamin D receptors. It has been shown that the use of vitamin D in mice increases the number of vitamin D receptors and the coating tissue becomes better integrated [25, 28]. The biological effect of vitamin D is applied via the nucleus receptors. These receptors are found in several tissues such as the liver, kidney, thyroid, adrenal gland, gastrointestinal tract, breast, and skin. Together, vitamin D and its receptors can negatively or positively affect the transcription of genes. Therefore, vitamin D is effective as a precursor in the differentiation and amplification of keratinocytes and restoring mucosal tissue of the vagina [29]. In addition, research has shown that squamous cell differentiation takes place in several steps, each of which is controlled by specific genes [30, 31].

The present review of existing literature shows that there are a few studies on the effect of vitamin D on vaginal atrophy and the efficacy of treatment has mostly been investigated under laboratory conditions using the Pap smear. No study has investigated the effect of vitamin D on clinical manifestations such as sexual function of postmenopausal women. On the other hand, the results of available studies have not been consistent. For example, Yildirmal et al. (2004) reported that the symptoms of vaginal atrophy in both Vitamin D treatment and control groups and that there were no significant differences between the groups [22]. On the other hand, Rad et al. (2014) reported significant differences after the end of 8 weeks among an intervention group with vitamin D vaginal suppository compared to the control group [24]. In another study, vitamin D was also used in combination with other treatments such as topical steroids and raloxifene [23], but the study did not provide conclusive evidence concerning the efficacy of vitamin D on vaginal atrophy. Other limitations of these studies were that some of them did not use a control group, random allocation, and blindness, and the duration of the follow-up of patients was short. Therefore, since there is no previous study concerning the clinical effect of the use vitamin D on women’s sexual function, and considering the strengths and weaknesses of previous studies, the proposed study is designed to evaluate the effect of vitamin D3 vaginal suppositories on the sexual function of postmenopausal women.

### Goals and hypotheses

The present study is based on the hypothesis that vitamin D vaginal suppository will have an effect on the sexual function of postmenopausal women by improving the symptoms of vaginal atrophy. As explained above, the possible mechanism might be due to the regulating and proliferative function of intracellular receptors of vitamin D in the basal and parabasal cellular layer in the epithelial tissue of the vagina [25–27]. The results of experiments in rats as well as a few approved clinical trials demonstrate that the use of vitamin D increases the number of vitamin D receptors and that the coating tissue is better integrated [24, 25, 28]. With this background in mind, the specific objectives of the proposed study include the:

- Comparison of sexual function in postmenopausal women within intervention, placebo, and control group, before, immediately after, and one and 2 months after the intervention;
- Comparison of sexual function in postmenopausal women between the intervention, placebo, and control groups immediately after, and one and 2 months after the intervention.

## Methods/ design

### Research setting and design

**Fig. 1:**
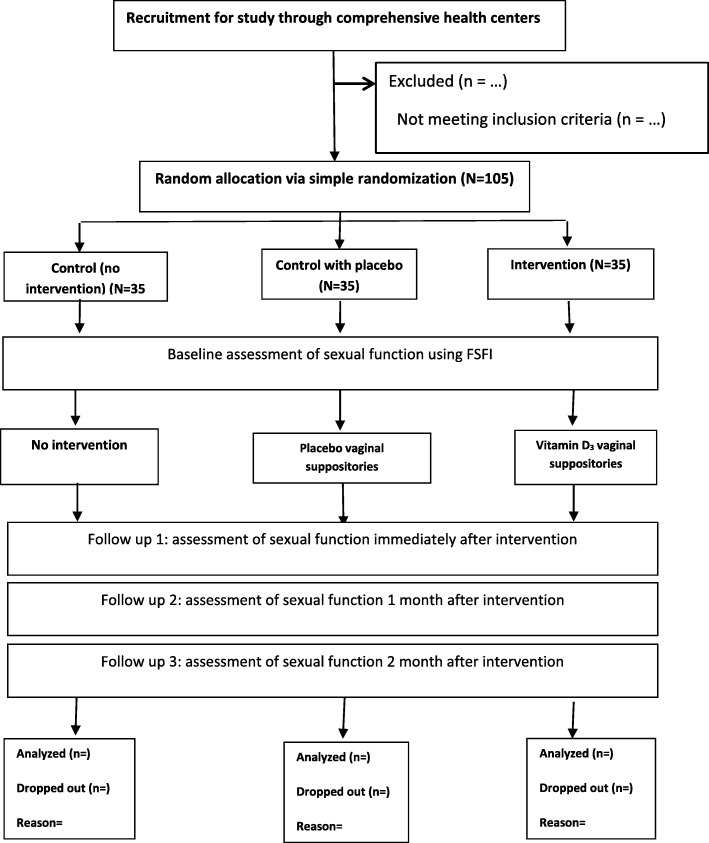
Consort diagram of study

The proposed study is designed as a randomized clinical trial with two parallel control groups of placebo and no intervention. Participants will be married women between the ages of 45 and 65 years referred to comprehensive health centers in the city of Buin Zahra, who meet the inclusion criteria. Buin Zahra is one of the cities of Qazvin province. This city consists of four areas (central, Dashtabi, Ramand and Shal). Figure 1 provide study flowchart.

### Participants

All postmenopausal women aged 45- to 65-years-old living in Buin Zahra district will be invited to participate in the study via their health care providers. Eligible participants from 25 urban and rural comprehensive health center affiliated to abovementioned four districts of Buin Zahra will be included in this study. The exclusion criteria will be (i) the presence of other endocrine diseases such as Cushing disease, diabetes, etc., (ii) using other vaginal drugs, (iii) having moderate to severe vaginal infection, (iv) undergoing hormone therapy, (v) having a stressful experience during the past 3 months, (vi) experiencing pelvic abnormalities, (vii) having recent surgery, and (viii) unwillingness to participate in the study. Also excluded will be those with absolute contraindications of vitamin D supplement including sarcoidosis, hyperphosphatemia, hypercalcemia, hyper-vitamin D levels, arteriosclerosis, kidney stones, and kidney disease that causes a reduction in kidney function.

### Sample size estimation

According to previous study by Çayan et al. [32], considering α = 0.05, power = 80%, moderate effect size 0f 0.6, the sample size for the research study was estimated to be 25 people for each group. Considering the attritional loss of 40% of the samples in the research process, the sample size for each group was calculated to be 35 people. The sample size calculation was performed according to primary outcome of study.

### Recruitment

To recruit the participants, eligible individuals will be selected on the basis of information in the health records of the comprehensive health centers in the city of Buin Zahra. A total of 105 eligible individuals will be invited to participate in the study. After screening the eligible individuals based on their health records, they will be called and invited for a visit in their comprehensive health care center. When they come for the screening visit, they will interviewed to assess eligibility criteria, introducing the project, its’ aim, their autonomy to participate in the study, confidentiality and anonymity of collected data. After signing the written consent, they will be randomized to study groups.

### Randomization

The participants will be randomly assigned into the study groups. Random allocation will be carried out using the simple randomization method, and assignment sequences will be written on paper before the start of the research as either A (suppository of vitamin D3 group), B (placebo suppository group), and C (control group without intervention). Randomization will be performed using random allocation software. The type of intervention will be written in accordance with the assignment sequence and will be enclosed within the opaque envelopes. Questionnaires will be also encoded in sequence. In this case, the questionnaire with the same code will be completed for the person receiving the code 1 intervention.

### Blinding

Due to having a control group which receives no intervention, blinding cannot be performed for all groups. But in try to blind intervention and placebo controlled group, vitamin suppositories and placebo suppositories will have the same color and shape and will coded as A and B by a pharmacologist not involved in the research team. The researchers will receive the suppositories with codes and will not know what A and B stands for with respect to the suppositories. Finally, after collecting questionnaires and analyzing data using SPSS version 25 software, the codes that belong to the groups will be determined.

### Intervention

After selecting the participants and random allocation to the groups, the participants’ sexual function will be assessed before the intervention. The participants in the treatment group will receive the vitamin D3 suppository and the placebo group will receive placebo suppositories identical the vitamin D3 suppositories in appearance. No intervention will be performed in the control group.

The use of vitamin D3 and placebo vaginal suppositories are consistent with the protocol used in the study by Rad et al. [24]. For 8 weeks, a single dose of 1000 unit vitamin D3 will be taken every night for the first 2 weeks, and every other night for the following 6 weeks. The reason for the choice of dosage and similar therapeutic protocol is because of the significant results reported in the improvement of para-clinical symptoms of vaginal atrophy based on the Pap smear. Since the results of this treatment have not been evaluated clinically, a similar dose is chosen for this study to examine the effect of vaginal vitamin D on sexual function.

### Storing vitamin D and placebo suppositories

The basis of the suppository is mono, di, and triglyceride called AM-15 suppository and synthesized by *Gattefosse France*. The base melting point is 34–36 degrees. Suppositories will be produced by melting and molding under the supervision of a pharmacist and will be produced by pharmaceutical experts from the pharmaceutical laboratory of the Faculty of Pharmacy of Mashhad University of Medical Sciences, Mashhad, Iran. Each suppository drug weighs 1 gram and contains 1000 units of vitamin D3. They have a sufficient mechanical strength and a smooth, uniform, whitewashed surface. After producing the suppositories, the correct amount will put in in tight plastic packages and placed in plastic containers at a temperature below 25 degrees, preferably kept in the refrigerator. Placebo suppositories will be made in exactly in the same way using Suppocire AM-15, with the exception that they lack the medication. They will be similar to pharmaceutical suppositories in terms of shape and color.

### Intervention program and patient education

During the first visit, the vaginal suppositories (without identifying the type of suppository, only having an identifier code inside the appropriate envelopes) will be given to the participant. The method and duration of use, as well as the follow-up time will be taught by the researcher to each participant. A phone number will be provided to ensure that the supplements are properly used and participants will reminded to keep track of the follow-up time. The researcher’s phone number will be shared with the participants to contact them if they have any questions about how to use the suppositories and/or if they experience any problems or signs of burning and itching following the use of the suppositories. As noted above, the total time period to take the suppositories for each postmenopausal woman will be 8 weeks, during which the investigator will monitor the use of the suppositories in the women by telephone or text-messaging (based on the convenience of the participant).

### Teaching the participants on how to use vaginal suppositories

Participants will be informed that (i) the suppository should be placed inside vagina before bedtime, but should not be inserted more than 3 inches inside the vagina, (ii) before placing the suppository in the vagina, their hands should be washed with water and soap, (iii) every night, only one suppository should be inserted into vagina, and (iv) the treatment schedule will be every night for 2 weeks and every other night for 6 weeks.

### Primary outcome measurements

Sexual function will be the primary outcome measure,. This will be assessed using the Female Sexual Function Index (FSFI). The FSFI comprises 19 questions of sexual function in six independent areas consisting of desire (two questions), arousal (four questions), lubrication (four questions), orgasm (three questions), sexual satisfaction (three questions) and pain from sex (three questions). Responses to the desire items are made on a six-point Likert scale from 0 (*never*) to 5 (*completely*) with a minimum score of 1 and a maximum score of 6. For other areas, responses are also made on a six-point Likert scale from 0 (*none*) to 5 (*completely*) with a minimum score of 0 and a maximum score of 6 [33]. The validity of the Persian version of this scale was evaluated and validated by Mohammadi et al. [34].

### Secondary outcomes

The secondary outcome measure will be participants’ satisfaction with the intervention.

### Baseline assessments

Evaluation of sexual function in postmenopausal women will be performed among all three groups at the beginning of the study and before the intervention.

### Follow-up assessments

Sexual function of women among all three groups immediately after the end of the intervention (duration of the intervention will be 8 weeks), and one and 2 months after the end of the intervention.

### Safety issues

Overall, the total intake dose of vitamin D3 will be 32,000 units during the 8 weeks of the intervention. The risk of poisoning with vitamin D is due to an overdose of vitamin D3 is 50,000 units per month (according to the Iran’s national guidelines) if administered over a long period of time. Given that the overall dose used in the proposed study is much lower than the level considered by the country’s guidelines, the probability of poisoning is low. In addition, to address concerns about the likelihood of poisoning with vitamin D3, the participants would be asked to withdraw taking supplemental vitamin D3 during the study. In addition, all symptoms of vitamin D3 poisoning will be taught to them and they will be asked to discontinue the supplements and inform the researcher if the signs of intoxication are detected. Any side-effects will be reported at the end of study.

### Data management and analysis

Data analysis and management will be performed using the SPSS software version 25. After collecting data, data entry will be performed on the dataset designed by ZS. The data entry accuracy and screening will be performed under the supervision of the ZA and MM. Initially, the normal distribution of data is investigated using the Kolmogorov-Smirnov test. In the case of normal distribution of data, the comparison of the mean scores of sexual function between the groups will be carried out using the repeated measurements ANOVA test. If the test is significant, a post-hoc test is used to determine the difference between the groups. Comparison of demographic and fertility characteristics of the women in the groups will be performed using appropriate statistical tests such as chi-square tests and one-way ANOVAs. If the assumption of normality is violated, transformation of data will be used. In most cases, transformation will result in normally distributed data and then parametric tests will be the main statistics employed. If not, a mixed effect model or non-parametric statistics will be used. A significance level of *p* < .05 will be used for statistical analysis.

## Discussion

Approximately one-third of postmenopausal women have reported painful intercourse (dyspareunia), lack of moisture, and lubrication [11]. Dyspareunia, vaginal dryness, and lack of lubrication are due to vulvovaginal atrophy [9, 10]. Estrogen therapy is one of the therapeutic methods for improving the symptoms of vaginal atrophy and dyspareunia in postmenopausal women [14]. However, due to the potential risks of postmenopausal hormone replacement therapy, systemic estrogen replacement therapy may not always be acceptable for women [8]. Concerns about the complications of estrogen therapy, including cardiovascular events, thromboembolism, breast cancer, and endometrial hyperplasia are among the most important reasons for the low acceptance of synthetic estrogen therapy and can lead women to utilizing alternative treatments for relieving menopausal symptoms [17].

Recent research has shown that vitamin D3 may also be useful in preventing vaginal atrophy. Vitamin D3 may play a role in regulating the growth and differentiation of the vaginal epithelium [6]. The possible mechanism for the effect of vitamin D on vagina is due to the presence of intracellular receptors of this vitamin in the basal and parabasal cellular layer in the tissue of the vagina. Because of these receptors in the vagina, vitamin D can play an important role in regulating and increasing the proliferation of epithelium cells in vagina [25, 26]. However, vitamin D receptors change during the menstrual cycle, which means that by stopping ovarian activity, the number of receptors are decreased [27]. Previous research has shown that squamous cell differentiation takes place in several steps, each of which is controlled by specific genes [30, 31]. Results of a clinical trial study on postmenopausal women showed that vitamin D vaginal suppositories could experimentally (according to the results of the participants’ Pap smear) improve dryness and cell proliferation of the vaginal mucosa in postmenopausal women [24]. Also, results of a cross-sectional study showed that the use of vitamin D supplements was effective in the maturation of vaginal cells [22].

As previous studies have shown a promising effect of vitamin D on vaginal cellularity and dryness, it might also be useful in improving sexual function. To best of the present authors’ knowledge, the proposed study is the first utilizing a randomized clinical trial design with two concurrent control groups of placebo and no intervention to examine the clinical effect of vaginal vitamin D on sexual functioning among premenopausal women. If vitamin D vaginal suppositories improve sexual function among premenopausal women with positive long-term effects and minimum side effects, the suppositories will be considered a safe complementary and alternative choice for alleviating sexual dysfunction among this group.

## Data Availability

Data and materials for the main research will be published along with publication of results.
